# Squamous Cell Carcinoma of the Tongue with Metastasis to Myocardium: Report of a Case and Literature Review

**DOI:** 10.1155/2019/1649580

**Published:** 2019-10-17

**Authors:** Ali Shafiq, Fatima Samad, Eric Roberts, Jonathan Levin, Ubaid Nawaz, A. Jamil Tajik

**Affiliations:** ^1^Aurora Cardiovascular Services, Aurora Sinai/Aurora St. Luke's Medical Centers, Milwaukee, WI, USA; ^2^BayCare Clinic Radiology, BayCare Health System, Green Bay, WI, USA; ^3^Department of Hematology and Oncology, Vince Lombardi Cancer Clinic, Aurora BayCare Medical Center, Green Bay, WI, USA

## Abstract

This is a case of a 43-year-old man who in 2014 was diagnosed with oral squamous cell carcinoma involving the tongue. He underwent extensive surgery that involved right tongue cancer resection and reconstruction with a free flap graft from his right forearm. He then was started on chemotherapy and radiation. Surveillance computed tomography in December 2016 showed a cardiac lesion in the left ventricular apex, which was confirmed by further echocardiography and cardiac magnetic resonance imaging. A biopsy of the mass revealed metastatic squamous cell carcinoma. He was deemed to not be a surgical candidate and continued on palliative chemotherapy. The patient had a very poor prognosis and eventually succumbed to the disease, highlighting the importance of surveillance imaging in such cases. A high index of suspicion on the part of the physician is needed to help in the early identification of these patients.

## 1. Introduction

Oral cancer is the 6th most common cancer in the world, with 9 out of every 10 oral cancers being oral squamous cell carcinomas (OSCCs). The tongue is involved in more than 50% of OSCCs [1, 2]. Although OSCCs may frequently metastasize to regional areas, distant metastasis—especially secondary involvement of the myocardium—is extremely rare. Most of these cases are generally diagnosed only at autopsy given that many patients are asymptomatic at the time of initial diagnosis. The risk factors most commonly associated with OSCCs are alcohol use, tobacco use, betel quid (combination of the betel leaf, areca nut, and lime), and female sex [2]. This is the main reason that OSCCs are prevalent in countries where there is frequent use of betel quid, such as on the Indian subcontinent. However, there has been a dramatic increase in the rate of OSCCs in the US over the last 30 years [3]. The most commonly cited reason for this rise is an increase in infection with high-risk human papilloma virus (HPV) [4]. We present a rare case of a young male diagnosed with OSCC, which, despite aggressive surgery and chemotherapy, eventually metastasized to his heart.

## 2. Case Presentation

A 43-year-old man with a history of leukoplakia underwent biopsy of his oral mucosa in 2010; it revealed moderate to severe dysplasia. He remained asymptomatic until 2014 when he felt a mass in his tongue. A computed tomography (CT) scan of the head and neck showed a density in the right tongue with no cervical lymphadenopathy. Biopsy of the tongue revealed moderately differentiated squamous cell carcinoma (SCC). A staging positron emission tomography (PET) scan demonstrated evidence of ipsilateral cervical lymph node involvement. He underwent tracheostomy, right neck dissection, right tongue cancer resection, and reconstruction with a free flap graft from his right forearm. Pathology revealed a 3 cm, invasive, well-differentiated SCC of the keratinizing subtype. The patient received 2 months of chemotherapy with cisplatin and radiation. A PET scan was done in May 2015 that showed complete remission.

**Figure 1:**
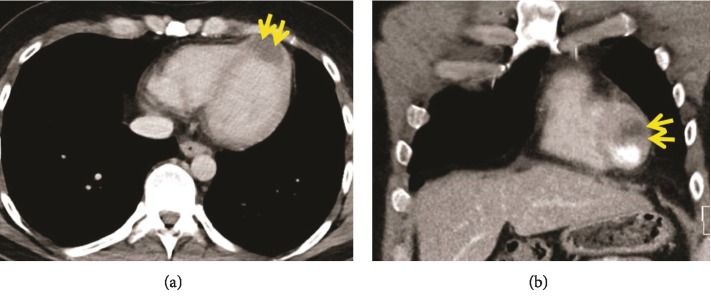
(a, b) Chest computed tomography with contrast shows a mass in the apex of the left ventricle (arrows).

**Figure 2:**
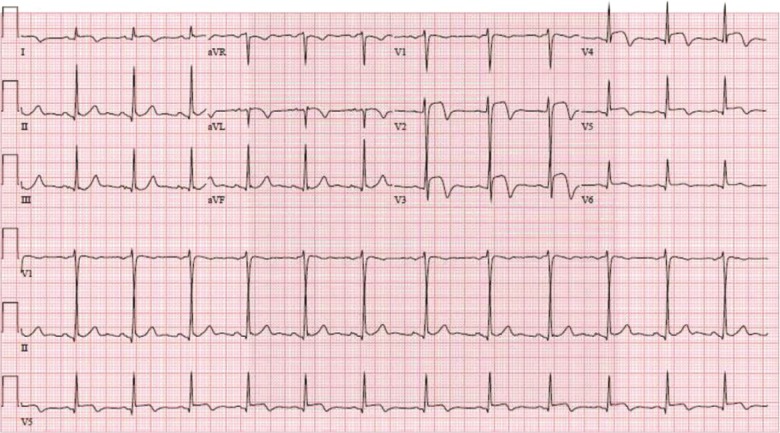
Electrocardiogram shows ST elevations in the anterior and lateral leads.

**Figure 3:**
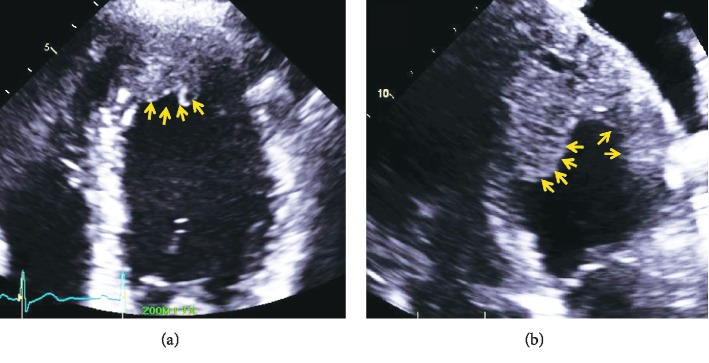
(a) Transthoracic echocardiogram shows a 4.6 × 2.8 cm mass (arrows) infiltrating the apical, antero-septal-lateral wall segments. (b) Transthoracic echocardiogram shows interval progression of the mass (arrows).

**Figure 4:**
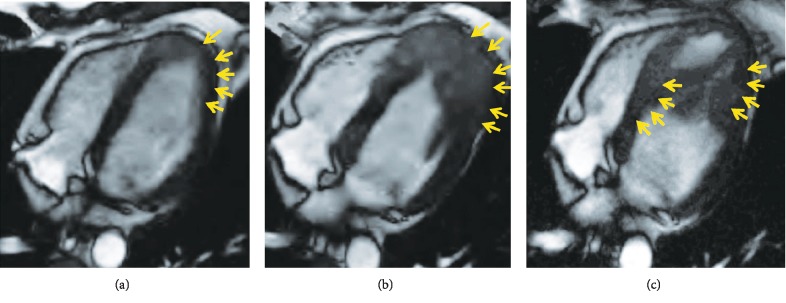
(a) Cardiac magnetic resonance imaging (MRI) demonstrates a 3.3 × 4.2 cm infiltrating lesion (arrows) within the apex of the left ventricle without early or delayed enhancement. (b) Cardiac MRI shows interval progression of the mass (arrows). (c) Cardiac MRI shows the infiltrative tumor mass involving the left ventricular myocardium with features suggesting central necrosis (arrows).

**Figure 5:**
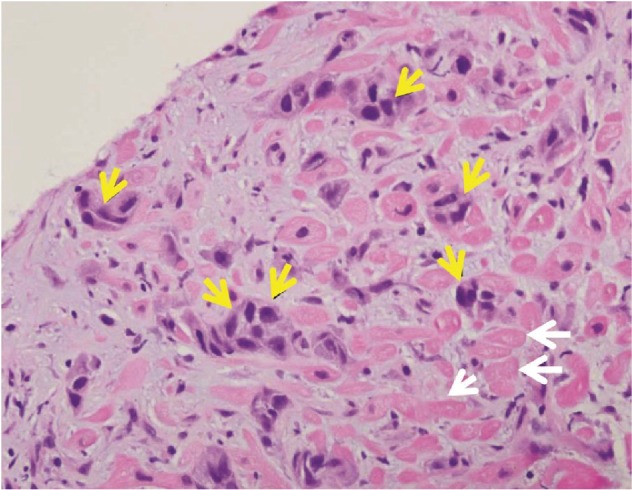
Pathology slide of right ventricular myocardial tissue shows pleomorphic malignant tumor cells (yellow arrows) and cardiac myocytes (white arrows).

**Figure 6:**
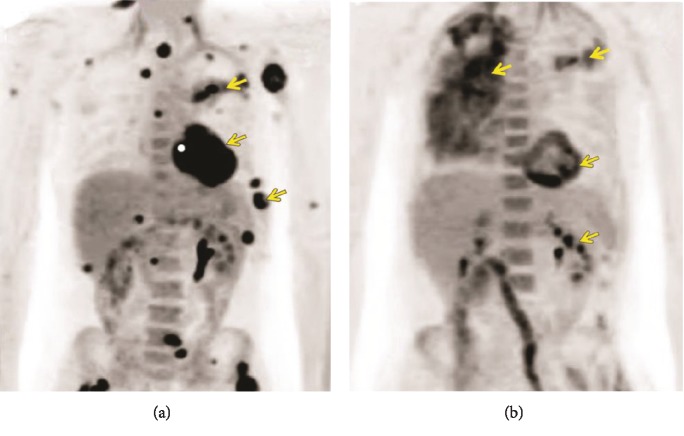
(a) Fluorodeoxyglucose positron emission tomography/computed tomography (FDG PET/CT) shows development of widespread hypermetabolic metastases (arrows). (b) FDG PET/CT shows more widespread metastasis involving the right lung and adrenal glands (arrows).

A surveillance CT scan done 1 year later, in May 2016, showed left lung lesions suspicious for metastatic disease, and bronchoscopy confirmed SCC of these lung lesions. He then underwent chemotherapy with 2 cycles of paclitaxel, carboplatin, and radiation. A repeat PET scan in September 2016 showed complete response, and the patient decided to proceed with observation. Another surveillance CT scan in December 2016 showed a cardiac lesion in the left ventricular (LV) apex (Figure 1). The patient was referred to our specialized cardiomyopathy clinic. In the clinic, the patient's physical examination was unremarkable but his electrocardiogram (ECG) showed ST elevations in the anterior and lateral leads suggestive of myocardial injury (Figure 2). He underwent a comprehensive transthoracic echocardiogram, which showed a 4.6 × 2.8 cm mass infiltrating the apical anteroseptal and anterolateral wall segments (Figure 3). The mass had an abnormal texture with less echodensity than the adjacent LV myocardium and was highly suspicious for metastatic disease. The patient underwent a cardiac magnetic resonance imaging (MRI) scan, which demonstrated a 3.3 × 4.2 cm infiltrating lesion within the apex of the LV without early or delayed enhancement (Figure 4(a)). The patient was referred for a right ventricular echocardiogram-guided myocardial biopsy. The pathology immunohistochemical stains (p40 and CK5/6) were consistent with myocardial involvement by metastatic SCC (Figure 5). The patient was started on palliative immunotherapy treatment with pembrolizumab. A follow-up cardiac MRI done 2 months later showed a substantial increase in the size of the mass as well as extension into the right ventricular apex (Figure 4(b)). A repeat PET scan done in March 2017 showed widespread metastasis (Figure 6(a)). The patient's treatment was switched to palliative combination chemotherapy with 5 fluorouracil, carboplatin, and cetuximab, to which he had a very good partial response. A repeat cardiac MRI done in July 2017 showed some improvement in the overall size of the cardiac mass. However, a cardiac MRI done in December 2017 showed interval progression of the infiltrative tumor mass involving the LV myocardium, with features suggesting central necrosis (Figure 4(c)). A follow-up PET scan showed multiple new metastatic lesions (Figure 6(b)). The patient was admitted to the hospital in February 2018 with worsening dyspnea and acute hypoxic respiratory failure. An echocardiogram showed interval progression of metastasis to the left and right ventricular cavities (Figure 4(c)).

## 3. Discussion

After the initial diagnosis of the OSCC, these patients usually have routine surveillance for the identification of any metastasis, but it is extremely rare to have secondary involvement of the myocardium. Through literature review, we found only 10 other patients (Table 1) who were found with an antemortem diagnosis of OSCC metastasis to the heart. We therefore present this unique case to raise awareness of this complication.

Among the previously reported cases, most patients were male and the patient age ranged from 36 to 73 years. Dyspnea, with or without congestive heart failure, appeared to be a frequent symptom at the time of diagnosis of the cardiac tumor. Only 1 patient [6] presented with typical angina, with symptoms attributed to the compression of her coronary arteries as a result of the anterior position of the tumor. In 2 patients, there were either no symptoms or nonspecific symptoms such as fever [11] and cardiac metastases were found incidentally on surveillance CT scans (similar to our patient). Although no specific ECG findings were reported, ST segment changes were noted in several of the patients (Table 1). Echocardiography was the cornerstone diagnostic modality to show the cardiac mass, and pericardial effusion appeared to be present in many of these patients [7, 8, 10, 11]. Cardiac MRI was used to define the extent of the cardiac mass in a few of the reported cases, as well as in our patient (Table 1). Surgical resection was attempted in a few of these patients; however, in our case, owing to extensive myocardial involvement, surgical resection was considered high risk and it was felt it would result in incomplete resection. Furthermore, given that it was not producing any degree of midventricular or outflow obstruction, palliative treatment was chosen and instituted.

Owing to the rare nature of cardiac involvement from primary OSCCs, routine cardiac imaging is usually not undertaken, often delaying this diagnosis. Surveillance CT scans often are done in patients with a primary malignancy and may help to unmask such rare cardiac involvement. Because of the variability in symptom presentation, a high index of suspicion on the part of the physician is needed to help in the early identification of these patients. Echocardiography supplemented with surveillance CT scan should be able to detect early lesions and allow better management strategy, including surgical excision.

## Figures and Tables

**Table 1:** Comparison of characteristics between cases reported with oral squamous cell carcinoma with metastasis to the heart.

Variables	Martell et al. [5]	Werbel et al. [6]	Rivkin et al. [7]	Schwender et al. [8]	Onwuchekwa and Banchs [9] (patient 1)	Onwuchekwa and Banchs [9] (patient 2)	Hans et al. [10]	Nagata et al. [11] (patient 1)	Nagata et al. [11] (patient 2)	Shimoyama et al. [12]	Shafiq et al. (current study)
Age (y)	60	61	57	73	45	36	54	59	69	71	43
Sex	Male	Female	Male	Female	Female	Female	Male	Male	Male	Male	Male
Primary cancer	SCC of retromolar trigone	SCC of base of tongue	SCC of base of tongue	Buccal SCC	SCC of tongue	SCC of tongue	SCC of base of tongue	SCC of tongue	SCC of left palate	SCC of tongue	SCC of tongue
At time of diagnosis of cardiac mass											
Symptoms	Dyspnea and edema	Angina	Lower extremity edema	Weakness and dyspnea	Syncope	Palpitations and dyspnea	Dyspnea and hemoptysis	Fever	None	Ulceration of oral cavity	Incidental
ECG findings	Atrial fibrillation	ST depressions with T wave inversions	Atrial fibrillation and ST elevations V2-V6	Atrial fibrillation	Normal sinus rhythm	ST elevations anterolateral leads	Right bundle branch block	Normal	Right bundle branch block and Q wave	ST elevations I, aVL, and V5-V6 and ST depressions II, III, and Avf	ST elevation anterior and lateral leads
Echocardiographic findings	Large mass in RV	Anterior mediastinal mass compressing RV outflow tract plus mass in right atrium	Pericardial effusion and RV mass	Pericardial effusion and echodense adherent lesions of anterior pericardium	Large mass at base of RV	Large mass infiltrating anteroseptal LV	Pericardial effusion and hyperechogenic mass involving RV	Pericardial effusion and mass in left atrium	Pericardial effusion and right atrial mass	Multiple echodense masses in LV	Mass in LV apex
Cardiac MRI	Yes	No	Yes	No	No	No	No	No	Yes	No	Yes
Pathology of cardiac mass	Metastatic, poorly differentiated SCC	Grade I SCC	Moderately differentiated SCC	Metastatic SCC	Biopsy not done	Biopsy not done	Biopsy not done	SCC	N/A	SCC	SCC
Treatment	Palliative radiation and chemotherapy	Surgical resection attempted but unsuccessful	Palliative chemotherapy	Developed septic shock and died	Palliative radiation of brain metastasis	Palliative treatment	Palliative treatment	Surgical resection of mass but died 3 weeks after surgery	Refused treatment and died 3 weeks later	Palliative radiation and chemotherapy	Palliative chemotherapy

ECG: electrocardiogram; LV: left ventricular; MRI: magnetic resonance imaging; RV: right ventricular; SCC: squamous cell carcinoma.
